# Real-space imaging of photo-generated surface carrier transport in 2D perovskites

**DOI:** 10.1038/s41377-025-01758-5

**Published:** 2025-03-18

**Authors:** Lijie Wang, Wentao Wu, Jie Yang, Razan Nughays, Yifan Zhou, Esma Ugur, Xi Zhang, Bingyao Shao, Jian-Xin Wang, Jun Yin, Stefaan De Wolf, Osman M. Bakr, Omar F. Mohammed

**Affiliations:** 1 Advanced Membranes and Porous Materials Center (AMPM), Division of Physical Science and Engineering, King Abdullah University of Science and Technology (KAUST), Thuwal, Saudi Arabia; 2 Key Laboratory of Material Physics, Ministry of Education, School of Physics, Zhengzhou University, Zhengzhou, China; 3 Department of Applied Physics, The Hong Kong Polytechnic University, Kowloon, Hong Kong SAR China; 4 KAUST Solar Center (KSC), King Abdullah University of Science (KAUST), Thuwal, Saudi Arabia; 5 KAUST Catalysis Center (KCC), King Abdullah University of Science and Technology (KAUST), Thuwal, Saudi Arabia

**Keywords:** Electronics, photonics and device physics, Optical techniques

## Abstract

In layered two-dimensional (2D) perovskites, the inorganic perovskite layers sandwiched between cation spacers create quantum well (QW) structures, showing large exciton binding energies that hinder the efficient dissociation of excitons into free carriers. This leads to poor carrier transport properties and low-performance light-conversion-based devices, and the direct understanding of the underlying physics, particularly concerning surface states, remains extremely difficult, if not impossible, due to the challenges in real-time accessibility. Here, we utilized four-dimensional scanning ultrafast electron microscopy (4D-SUEM), a highly sensitive technique for mapping surface carrier diffusion that diverges from those in the bulk and substantially affects material properties. We directly visualize photo-generated carrier transport over both spatial and temporal dimensions on the top surface of 2D perovskites with varying inorganic perovskite layer thicknesses (*n* = 1, 2, and 3). The results reveal the photo-induced surface carrier diffusion rates of ~30 cm^2^·s^-1^ for *n* = 1, ~180 cm^2^·s^-1^ for *n* = 2, and ~470 cm^2^·s^-1^ for *n* = 3, which are over 20 times larger than bulk. This is because charge carrier transmission channels have much wider distributions on the top surface compared to the bulk, as supported by the Density Functional Theory (DFT) calculations. Finally, our findings represent the demonstration to directly correlate the discrepancies between surface and bulk carrier diffusion behaviors, their relationship with exciton binding energy, and the number of layers in 2D perovskites, providing valuable insights into enhancing the performance of 2D perovskite-based optoelectronic devices through interface engineering.

## Abstract (web summary)

Using 4D-SUEM, we revealed photo-induced surface carrier diffusion in 2D perovskites, with rates far exceeding bulk values, driven by broader carrier transmission channels, highlighting the crucial role of surface dynamics.

## Graphical abstract

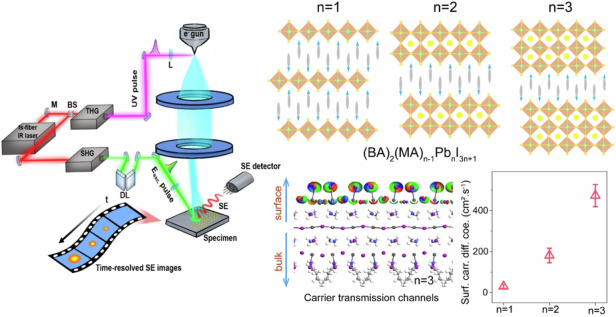

## Introduction

Efficient charge carrier transport is pivotal for the performance of optoelectronic materials^1^, and its understanding traditionally relies on indirect spectroscopic methods such as photoluminescence (PL) quenching^2,3^, terahertz photoconductivity^4^, or time-of-flight techniques^5^, determining charge carrier mobilities or diffusion constants^6^. However, these methods describing transport in spatially homogeneous materials, often yield incongruent results, with values varying significantly for the same material system, possibly due to probing different excited-state processes or sample statuses by the various techniques^7^. Recent advancements in transient absorption microscopy (TAM) provide an alternative by directly tracking carrier transport in space and time^8–11^, mapping carrier densities with excellent time and spatial resolutions. Nevertheless, the combination of microscopy techniques with ultrafast spectroscopic methods, where the photon probe primarily detects bulk information in the range of several hundred nm or even μm, lacks access to surface states that can notably impact charge carrier transport parameters in semiconductor materials.

Among these, 2D perovskites are considered one of the highly promising optoelectronic materials generated by slicing 3D perovskites by incorporating bulky organic spacer cations to separate the inorganic slabs^3,12,13^. This forms multiple quantum well (QW) structures, with the sandwiched octahedral layers that serve as the well depth and the organic cation layers acting as barriers^14^. The unique photoelectric characteristics of 2D perovskites provide a versatile platform for applications^15–17^, offering superior stability and wide compositional tailoring^18^. However, this structure results in an increased exciton binding energy, making the efficient dissociation of photo-generated excitons into free carriers difficult to achieve^19,20^. Besides, the van der Waals gap hinders carrier transport across adjacent QWs with different widths. On the other hand, increasing the number of inorganic octahedral slabs/layers leads to lower exciton binding energies^21^, although the excessive increase in these inorganic layers produces bulk-like characteristics, elevating the monomolecular recombination rate and the concentration or effectiveness of trap states^22,23^, in turn, reduces the carrier transport properties. Additionally, free carriers disassociated from certain higher-energy excitonic states, could potentially trap at the so-called layer-edge states^22,24^. In solar cells, surface/interface states are recognized as key factors in their performance yet remain poorly understood. Therefore, identifying optimal parameters for carrier surface transport in 2D perovskites becomes urgent for their large-scale applications.

In this work, we conducted 4D scanning ultrafast electron microscopy (4D-SUEM), a method developed to have the sensitivity for spatiotemporal imaging of carrier dynamics following photoexcitation at material surfaces. Previously this technique was applied successfully to capture entire sequences of charge carrier generation, transport, and recombination in hydrogenated amorphous silicon^25^, 2D MoS_2_^26^, and at a silicon p-n junction^27^. Note that in 4D-SUEM, a primary electron beam, produced by a delayed UV excitation pulse (~345 nm), is utilized to generate secondary electrons (SEs) from the specimen’s surface, which enables the probing of local carrier (electron/hole) density rather than excitons on surfaces and interfaces^28–31^, and allows us to address the charge carrier surface diffusion in 2D perovskites with varying QW thicknesses. Our investigation reveals that 2D perovskites with dimensionalities *n* = 2 and 3 demonstrate a more efficient exciton dissociation rate and smaller charge carrier effective masses, thereby exhibiting significant advantages over *n* = 1 case in terms of diffusion distances, which aligns well with the results obtained from ultrafast spectroscopic analyses and density functional theory (DFT) calculations. Particularly, we identified a greater concentration of charge carrier transmission channels on the top surface compared to the bulk, especially evident with an increasing number of inorganic perovskite layers. Such distribution properties interpret the distinctive surface-to-bulk carrier transport discrepancies and underscore the importance of surface behaviors in the application of 2D perovskite materials.

## Results

**Fig. 1:**
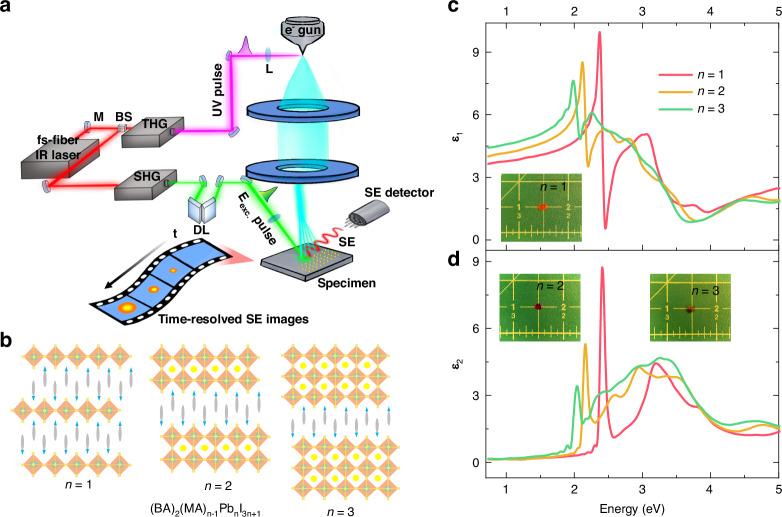
**Schematic diagram of 4D-SUEM setup and fundamental optical and structure properties of 2D perovskites. a** Schematic illustration of probing photo-generated charge carriers via 4D-SUEM. The entire setup comprises a modified SEM with the integration of an ultrafast fs-fiber laser system. Beams generated through second harmonic generation (SHG) and third harmonic generation (THG) are directed toward the sample surface for optical excitation and the electron gun inside of the SEM. Pulsed electrons are used to probe the surface changes upon excitation and generate time-resolved secondary electron (SE) images. **b** Schematic structures of 2D halide perovskite ((BA)_2_(MA)_n-1_Pb_n_I_3n+1_) with different numbers of inorganic layers (*n* = 1, 2, 3). **c**, **d** The real (ε_1_) and imaginary (ε_2_) parts of the dielectric function of 2D perovskite crystals were calculated based on fits from the variable angle spectroscopic ellipsometry data. The inset shows photos of the prepared 2D perovskite crystals

A schematic illustration of the probing process for photo-generated charge carriers using 4D-SUEM on the layered 2D perovskites is shown in Fig. 1a. The entire setup comprises a modified SEM with the integration of an ultrafast fs-fiber laser system, and the details of the setup is given in the experimental section. Upon laser irradiation, charge carriers are generated at each layer near the surface and rapidly dispersed to the surrounding regions along the planar directions. Typically, the generated carriers exhibit a Gaussian-like distribution in both space and time, with the most intense part in the center of the laser spot. It is worth noting that in 4D-SUEM, we detect secondary electrons that are sensitive to the uppermost layers within a few nm (1–5 nm) to scan the sample surface, detecting the dynamic behavior of free carriers, specifically electrons and holes, rather than excitons. Generally, we eliminate the background signal by subtracting a reference image at a far negative delay time, resulting in contrast images where increased intensity (bright/positive) indicates electron accumulation, while decreased intensity (dark/negative) signifies increased hole concentration^25^. This approach ensures that the detected signal corresponds solely to variations in the local carrier density induced by laser excitation (see Fig. S1, the raw steady-state SUEM images of 2D perovskite surface).

Figure 1b illustrates the structure of the investigated layered 2D perovskite single crystals, (BA)_2_(MA)_n–1_Pb_n_I_3n+1_ with n = 1, 2, and 3. Here, BA denotes a long-chained monoamine organic cation spacer layer, with subsequent atomically thin negatively charged layers stacked via van der Waals forces, thus creating QW structures (Fig. S2). The value of n can be tailored through precise control of chemical stoichiometry, although achieving phase-pure materials at higher n values is often challenging due to phase separation^32^. It is noteworthy that within the same plane, an inorganic layer is offset by the displacement of one octahedral unit, forming what is referred to as the Ruddlesden–Popper structure^33,34^. Excitons govern the optical responses across all three structures, as demonstrated by the absorption and PL spectra. Variable angle spectroscopic ellipsometry data at three different angles were measured to extract the dielectric functions and the absorption coefficient of the three samples (Fig. 1c, d). Details regarding the fitting of the measured spectra and methods are provided in the experimental section and SI (see Note S1, and Fig. S3 for spectroscopic ellipsometry data). In Fig. S4, a notable increase in the bandgap (Eg) from ~1.95 eV to ~2.35 eV, is recorded as the value of n decreases from 3 to 1, which can be attributed to the quantum and dielectric confinement effects^35^. Furthermore, the excitonic peaks shift to higher energies, becoming sharper and more intense, particularly in the *n* = 1 sample, which is also correlated with the exciton emission peaks in the PL spectra (Fig. S5), suggesting stronger bound excitons and a greater concentration of oscillator strength within the exciton absorption band compared to the *n* > 1 QWs. In addition, XRD analyses were performed on the three prepared crystals and compared with simulations derived from standard single-crystal XRD data (Fig. S6), these above results clearly suggest the excellent quality and phase purity of the prepared samples.

**Fig. 2:**
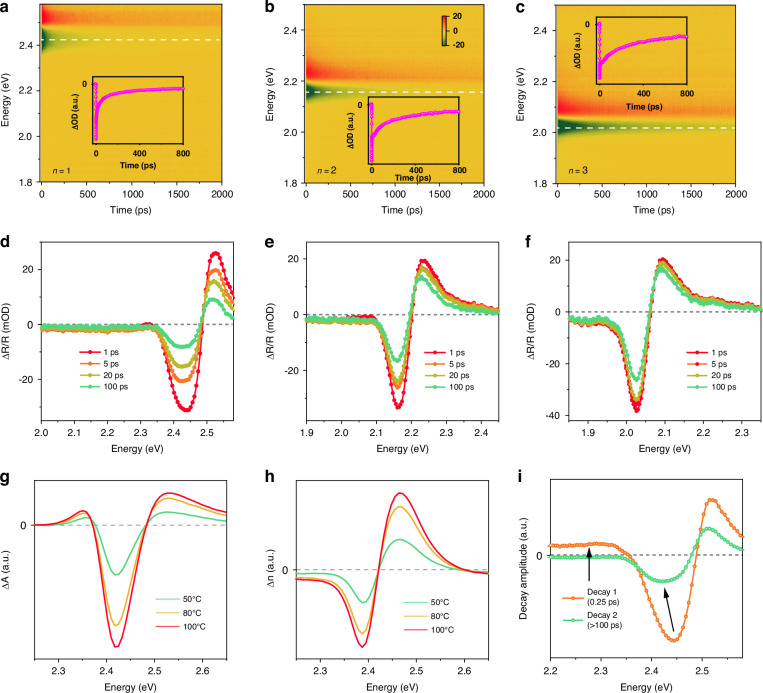
**Time-resolved optical spectroscopy results. a**–**c** Experimental transient reflectivity maps of *n* = 1, 2, and 3 samples probed in the visible spectral region upon 3.06 eV (405 nm). The inset shows the corresponding kinetic traces selected at the maximum negative peak position along with fitting. **d**–**f** Corresponding transient spectral traces of 2D perovskites with *n* = 1, *n* = 2, and *n* = 3 at 1, 5, 20, and 100 ps, respectively. **g** Temperature-induced differential absorption spectra in *n* = 1 sample, at 50, 80, and 100 ^o^C, respectively. **h** Temperature-induced differential refractive index in *n* = 1 sample. **i** Decay-associated spectra representing the initial (sub-ps), and long-term (>100 ps) evolutionary processes in the *n* = 1 crystal

We firstly performed transient reflectivity (TR) experiments on these three crystals, all under identical conditions with the same parameters. Figure 2a–c depict the TR maps of the *n* = 1, 2, and 3 samples under 3.05 eV (405 nm) excitation, respectively, while Fig. 2d–f presents the related spectral traces at different delay times, and the inset exhibits the corresponding kinetic traces selected at the minimum of negative peak positions along with fitting. We observed that the *n* = 1 crystal exhibits derivative like signal, which is positive above and negative below ~2.48 eV, these signal profiles resemble the ones observed in 3D perovskites^36^ where it was assigned to the bleaching of the excitonic feature caused by phase-space filling (PSF), along with a positive feature peaking at ~2.52 eV, ascribed to excited-state absorption (ESA) of the photo-generated charged species^37,38^ (see Note S2 in SI). Here we take the *n* = 1 sample as an example for calculating the reflectivity and absorption spectra from temperature-dependent spectroscopic ellipsometry (see Note S3). The resulting temperature-induced differential absorption (ΔA) and refractive index (Δn) are displayed as functions of energy and temperature in Fig. 2g and h. Additionally, a global lifetime analysis (GLA) was carried out with a time window of 0-100 ps, and the resulting decay-associated spectra (DAS) are presented in Fig. 2i. The DAS effectively separates the different contributions at specific time delays, providing information on the spectral profiles corresponding to different processes after photoexcitation^39^. Notably, the DAS1 curve resembles the temperature-induced changes in absorption (note the two positive wings on the high- and low-energy sides), while DAS2 aligns well with the temperature-induced changes in refractive index (noting the negative peak shift).

For *n* = 2 and 3 crystals, the TR spectra also display two primary features: a negative band centered around 2.16 eV (*n* = 2) and 2.03 eV (*n* = 3), accompanied by a positive ESA band peaking at 2.22 eV (*n* = 2) and 2.08 eV (*n* = 3). A comparison of the three spectra traces recorded at 1 ps is shown in Fig. S7. However, fitting the kinetics of the TR peaks with multi-exponential functions uncovers distinct decay lifetimes across these crystals (see Fig. 2 inset and Fig. S8). Rapid decay kinetics are observed for all three traces with similar timescales in the hundreds of fs regime (see the inset of Fig. S8, and τ_1_ values in Table S1,). This phenomenon has been reported in perovskite materials and is attributed to hot-hole cooling from deeper to the upper valence band (VB)^40^. Subsequently, for the crystal of *n* = 1, the TR kinetics measured at the negative peak demonstrate a slower decay with τ_2_ = 22.5 ± 4.1 ps and τ_3_ = 341 ± 55 ps, whereas the *n* = 2 and *n* = 3 cases display much longer lifetime constants of τ_2_ = 104.1 ± 8.2 and 152.2 ± 18.0 ps, and τ_3_ = 870 ± 89 and 1111 ± 160 ps, respectively (refer to Table S1). These results imply a slower electron-hole recombination rate in the *n* = 2 and *n* = 3 cases.

**Fig. 3:**
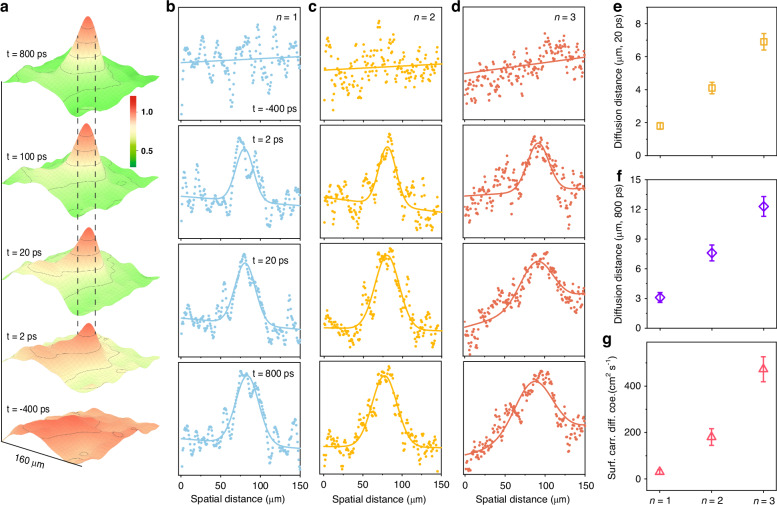
**Results on 2D perovskite from 4D-SUEM. a** Representative 3D color-coded maps illustrating the evolution of photo-induced electron transport in the *n* = 3 2D perovskite sample. These images are derived from the 4D-SUEM difference images, with a reference image at a far negative timescale. Each image corresponds to the average of 4 frames captured at identical time points and is processed using a 2D Gaussian function. Maximum intensities are normalized to 1 for comparison. **b–d** Spatial distance along the Y direction in the center of the laser-induced spots in the *n* = 1, 2, and 3 2D perovskite. The dots indicate the raw experimental measurements, while the solid lines depict fitted curves consisting of a linear component representing the background and a Gaussian function to represent the signal distribution, given by ${\rm{G}}\left(x\right)=\frac{1}{\sigma \sqrt{2\pi }}{e}^{-\frac{{x}^{2}}{2{\sigma }^{2}}}+{ax}+b$. The FWHM is calculated based on ${\rm{FWHM}}=2{\rm{\sigma }}\sqrt{2\mathrm{ln}(2)}$. **e**, **f**, Diffusion distances at ~20 and 800 ps of the three measured samples. We assume the spot at ~2 ps represents the laser footprint, and the diffusion is calculated by using half of the difference of FWHM (${({FWHM}}_{20,800{ps}}-{{FWHM}}_{2{ps}})/2$). **g** Estimated surface carrier diffusion coefficients for n values between 1 and 3. The error bars represent the upper and lower limit values

Figure 3a illustrates the representative 3D color-coded SUEM difference maps depicting the evolution of photo-induced electron transport in the *n* = 3 samples. Each image is an average of 4 individual images taken at identical time points to enhance the signal intensity and is filtered using a two-dimensional Gaussian function to smooth the signal, with the maximum intensities of peaks normalized to 1 for comparison. At negative time delays, the samples exhibit nearly flat contrast, however, upon the arrival of photon pulses and with increasing delay time, a distinct Gaussian-like signal emerges and grows. The dashed lines around the center of the peaks, serve as a reference for the growth and broadening of the peaks. Here we assume the image captured at ~2 ps represents the laser footprint due to the relatively large pulse duration and slow signal buildup process after excitation (see Fig. S9 for a standard test on Silicon sample), and later use it as a reference to estimate the diffusion distances at different time delays among the three samples. Figure S10 displays SUEM images before and after photoexcitation on the surface of the sample to confirm its stability under laser excitation. The dashed circle represents the laser footprint, and the different images clearly demonstrate the evolution of photo-generated electron diffusion on the surface (Fig. S11). The accurate transient response analysis is better highlighted by cutting the signal along either the X or Y axis through the peak center to extract a spatial profile that reflects the actual peak evolution in a Gaussian-like curve. This allows for fitting to obtain rich parameters, detailing the extent of peak broadening with time. Figure 3b–d shows the spatial distances along the Y direction at the midpoint of the laser-induced region for the three measured samples. The blue, yellow, and red dots in the graph reflect the original experimental data, whereas the solid lines show the fits with functions that include a linear component for the background and a Gaussian function for the signal distribution, as expressed by

$${\rm{G}}\left(x\right)=\frac{1}{\sigma \sqrt{2\pi }}{e}^{-\frac{{x}^{2}}{2{\sigma }^{2}}}+{ax}+b$$

The Full Width at Half Maximum (FWHM), which deduces the diffusion, is calculated based on $2{\rm{\sigma }}\sqrt{2\mathrm{ln}(2)}$. It is evident that the peak width notably increases until 800 ps for all three samples compared to that recorded at ~2 ps. The relative diffusion distance is calculated by taking half of the difference in FWHM (${({FWHM}}_{20,800{ps}}-{{FWHM}}_{2{ps}})/2$), and is plotted on the right side in Fig. 3e and f. These values of ~3.1, ~7.6, and ~12.3 μm have reasonable trend, but they differ from the reported carrier diffusion lengths measured by time-resolved PL (tr-PL), which predominantly represent bulk information, as photon probes have a much greater penetration depth than electron probes. The comparison between the reported values and ours is summarized in Table S2. The study by S. Jin et al.^41^ observed rather similar carrier transport distances in *n* = 2 and *n* = 3 samples, although the carrier diffusivity and lifetime are larger in the latter case. This was claimed to be due to exciton dissociation triggered by traps, leading to a long-lived, non-radiative state with spatially separated electrons and holes, and subsequently enabling charge transport through trap-assisted pathways. While Nie et al.^42^ reported progressively increasing diffusion distances with the number of layers, concluding that samples with completely 3D structures had the farthest diffusion distance, which contradicts the results with Herz et al*.*^23^ that the intermediate states of 2D perovskites between *n* = 1 and 3D have smaller charge-carrier recombination rates and longer transport distances.

For two dimension diffusion on the sample’s surface, the diffusion constant D is then given by refs. ^8,43^

$${\rm{D}}=\frac{{{{FWHM}/2}_{t}}^{2}-{{{FWHM}/2}_{2{ps}}}^{2}}{4t}$$

where ${FWHM}/2$ is half of the FWHM of Gaussian-like carrier distribution peaks at specific delay time. The estimated surface carrier diffusion coefficients under the used excitation fluence for *n* values between 1 and 3 are presented in Fig. 3g. The *n* = 1 sample exhibits the smallest values of ~30 cm^2^·s^-1^, which is reasonable as the transport is restricted within the QW plane, constrained by a short charge carrier lifetime and limited mobility. With an increasing number of perovskite layers, the values notably rise to ~180 cm^2^·s^-1^ for *n* = 2 and ~470 cm^2^·s^-1^ for *n* = 3. This trend correlates well with the lifetimes extracted from the TR measurements, the exciton dissociation efficiency^20^ (Fig. S12), and reported solar cell device performance^44^, and is inversely proportional to the exciton binding energy^18^ (Fig. S13). However, according to Shreetu et al.’s report^42^ with the tr-PL method primarily measuring the bulk transport, the *n* = 3 cases yield a carrier diffusion coefficient of approximately 18 cm^2^·s^-1^, which is more than ~20 times smaller than our findings detecting surface carrier diffusion. This significant difference could stem from surface states in semiconducting materials, which substantially influence material properties and will be discussed later. It should be noted that in SUEM experiments, the excitation wavelength is fixed while 2D perovskites with different layer thicknesses have different band gaps and absorption properties, meaning the *n* = 3 sample has the largest excess energy of photons, which could potentially impact carrier transport behaviors. Nevertheless, the trend of diffusion distances in the three crystals aligns well with reported cases. The main significance of this study lies in the distinctive surface-to-bulk carrier transport discrepancies.

**Fig. 4:**
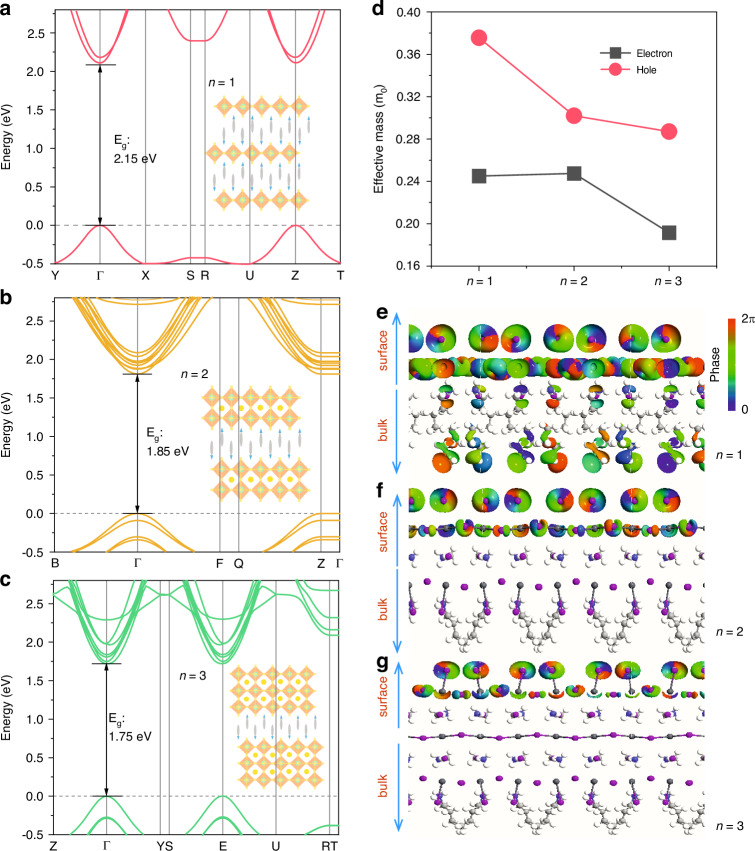
**Calculated band structures and impact of electron transmission eigenstates on transport properties. a**–**c** Electronic band structures of 2D perovskites corresponding to *n* = 1, 2, and 3, respectively, derived from calculations performed using GGA/PBE+vdW method. **d** The effective mass of electrons and holes in the corresponding 2D perovskites. **e**–**g** Transmission eigenstates at the Г point at the Fermi level of the three types of samples, the top side represents the top surface of the sample layers, while the bottom side faces toward the bulk. The intensity of the color indicates the distribution of transmission channels filled within the sample from the surface to the bulk. The isovalue of the transmission eigenstate is 0.15 (Å^3^·eV)^-1/2^

To understand the carrier transport properties and surface states in 2D perovskite materials, we performed DFT calculations to analyze the electronic band structures and determine effective masses. As shown in Fig. 4a–c, all 2D perovskites are direct-bandgap semiconductors, exhibiting a clear 2D electronic structure with dispersive bands along the layer in-plane direction (i.e., for *n* = 1, Γ → Y and Γ → X) and flat bands along the out of-plane stacking direction (i.e., Γ → Z). The calculated band gaps are slightly smaller than the experimental ones, which aligns with the well-documented tendency of the GGA/PBE method to undervalue the band gaps of perovskite materials^45^. Within the layer direction, the calculated effective masses for both holes and electrons range from 0.15 *m*_0_ to 0.40 *m*_0_ (Table S3). With increasing the number of layers, there is a trend of decreasing average effective masses, from 0.25 *m*_0_ to 0.19 *m*_0_ for electrons and from 0.38 *m*_0_ to 0.29 *m*_0_ for holes, as depicted in Fig. 4d. This suggests higher carrier motilities in 2D *n* = 2 and 3 perovskites as compared to the *n* = 1 case, which is consistent with the proportional relationship between oscillator strength and exciton binding energy, along with the calculated exciton dissociation efficiency of ∼85% for n = 3, ∼50% for *n* = 2, and ∼4% for *n* = 1 (Fig. S12).

However, the above mentioned calculations pertain to the ensemble level rather than the local region on the surface of the materials, we next calculated charge carrier transmission eigenstates at the Γ point at the Fermi level for 2D perovskites, mapping the distribution of carrier transmission channels from the top surface (upper side) toward the bulk (lower side) of the three studied samples shown in Fig. 4e–g. It is evident that for the n = 1 2D perovskite, the carrier transmission channels almost entirely populate regions from the surface to the bulk, with notable pathways extending into the organic linkers, despite a seemingly higher concentration of channel distributions on the surface. This configuration suggests that photo-generated electrons and holes can readily undergo mutual scattering and become trapped within the bulk. However, with increasing layer thickness of the 2D perovskites to *n* = 2 and *n* = 3, the transmission channels become more concentrated on the top side, facilitating the smooth spreading of charge carriers along the surface plane. The calculated transmission coefficient at the Fermi level indicates that as the perovskite layer number increases, the coefficient increases from ~0.5 (*n* = 1), to ~0.7 and ~0.9 for *n* = 2 and 3, respectively (see Fig. S14). Additionally, the transmission gaps near the Fermi level for the *n* = 2 and 3 samples are much narrower compared to the *n* = 1 case, which is in good consistency with the layer-dependent E_g_. These observations rationalize that 2D perovskites, especially the *n* = 3 crystal, demonstrate greater carrier transport possibilities, particularly on the top surface compared to the bulk. This indicates heterogeneous charge carrier transport characteristics, elucidating why conventional time-resolved spectroscopic and microscopic methods may fail to discern or even yield large discrepancies in results.

## Discussion

In summary, we have successfully visualized the transport of photo-generated charge carriers on 2D perovskite materials at ultrafast timescales using SUEM, which has the unique surface-sensitive capability. By utilizing SUEM, we are able to accurately explore carrier diffusion in the local region of a material’s top surface following photoexcitation. This method provides a clear distinction from traditional bulk or ensemble spectroscopic techniques, which may not accurately distinguish surface-to-bulk states in 2D perovskites. Our research has revealed that surface carrier diffusion coefficients are significantly larger, more than 20 times, than those reported for bulk materials. This discrepancy can be attributed to the distinct surface states of 2D perovskites, which feature significantly higher concentrations of charge carrier transmission channels on the top surface compared to the bulk. This work not only demonstrates the potential of combining spatially and temporally experimental tools such as surface-sensitive SUEM and ultrafast spectroscopy, alongside advanced DFT calculations to gain new insights into the surface transport of photo-generated carriers in low-dimensional nanostructures, but also reveals the great promise of 2D perovskites in terms of surface/interface engineering and structure programmability for their optoelectronic applications.

## Materials and methods

### Single crystal preparation

The 2D perovskite single crystals were grown from aqueous solutions using a well-established crystallization approach involving the controlled cooling of a saturated solution^34^. Specifically, PbI_2_ powder (20 mmol) was dissolved in a solution composed of 20 mL of aqueous HI (57 wt.%) and 3 mL pf aqueous H_3_PO_2_ (50 wt.%) in an 80 mL covered vial. The mixture was brought to a boil under continuous magnetic stirring for approximately 7 minutes in a fume hood, producing a bright yellow solution. Subsequently, CH_3_NH_3_I and n-CH_3_(CH_2_)_3_NH_2_ were gradually added into the hot solution at different molar ratios (0 and 40 mmol for *n* = 1, 10 and 15 mmol for *n* = 2, 13.34 and 6.66 mmol for *n* = 3). Stirring and heating continued until the solution became clear. The vial was then tightly sealed, wrapped with parafilm, and placed in an oven at 343 K, where the hot, oversaturated solution was cooled at a controlled rate of 2 K per day. Plate-shaped crystals began to form in the vial as the solution cooled, with their size gradually increasing as the temperature continued to decrease, and the crystal thickness could be controlled by modulating growth duration. The entire process was conducted under ambient conditions, and the crystals were dried by gently blowing dry nitrogen over the surface for around 10 minutes. In addition, the top layer of the single crystal was mechanically peeled away to expose a fresh, clean surface for characterization. This was accomplished by adhering tape to both sides of the crystal and gently peeling it away to achieve an even removal.

### Spectroscopic ellipsometry measurements

Spectroscopic ellipsometry measurements were conducted with an M-2000 DI device (J. A. Woollam, USA), covering the wavelength range of 193–1690 nm. The sample was analyzed at three incidence angles (65°, 70°, and 75°), with two focusing lens that has the capability to measure ultra-small samples. The VASE data was fitted using an isotropic “B-spline” mode^46^, allowing for the determination of the absorption coefficients and refractive indexes, the Complete EASE 6.51 software suite was used for data analysis, and the raw and fitted results are shown in Fig. S3.

### Ultrafast spectroscopy measurements

The TR measurements utilized an Ultrafast Systems Helios pump–probe setup for broadband visible probing under tunable excitation. A 1 kHz 150 fs/7 mJ 800 nm laser pulse was split to generate the pump and probe beams. The pump pulses, spanning ~350–700 nm, were produced via a nonlinear optical parametric amplifier (SpectraPhysics). The probe beam was generated by focusing the residual 800 nm pulse into a 2 mm CaF_2_ plate. During all measurements, the pump fluence at 405 nm (3.06 eV) was around 50 μJ·cm^-2^, with an estimated uncertainty of ~10% arising from variations in beam spot size and laser power. The pump power was monitored shot-by-shot using a calibrated photodiode, allowing for normalization of the data relative to the pump power. Notably, the white light probe beam was divided into two parts, with one serving as a reference to substantially enhance the signal-to-noise ratio.

### SUEM measurements

A femtosecond Clark-MXR pulsed laser operating at 1030 nm was integrated into a modified QUANTA 650 scanning electron microscope. The laser beam, with a 6.25 MHz repetition rate, was directed via mirrors to a 60/ 40 beam splitter, dividing it for third and second harmonic generations. The resulting 515 nm green beam was focused onto the sample within the electron microscope to induce excitation, while the UV component was aimed at the electron gun tip to produce pulsed electrons, replacing the traditional thermal electron generation. An electronically controlled delay stage adjusted the pump pulse timing relative to the probe. The pump beam footprint was determined by performing beam-on/beam-off measurements and validated against a reference silicon sample, and the pump fluences used are similar to the spectroscopic measurements which is ~60 μJ·cm^-2^ (equivalent to a carrier density of ~1.8 × 10^18^ cm^-3^), to avoid sample damage that still ensures data quality. The samples were placed flat on conductive tape within the SEM chamber, and a clean surface area was carefully selected under the electron microscope. An important consideration in this step is to select a sample with a relatively large size and sufficient flatness. The top layer of the crystal samples was removed by adhering tape to both sides and gently peeling it away to expose a fresh, clean surface, and the sample was positioned flat on the stage to avoid any angular discrepancy between the sample surface and the stage. Additional details regarding the experimental setup can be found in ref. ^47^.

### DFT calculations

Density functional theory (DFT) computations were performed utilizing the projector-augmented wave (PAW) method, as integrated within the Vienna Ab initio Simulation Package (VASP) code^48,49^. The calculations utilized the generalized gradient approximation (GGA) together with the Perdew-Burke-Ernzerhof (PBE) exchange-correlation functional. To account for van der Waals (vdW) interactions, the DFT-D3 scheme by Grimme with zero damping was applied. Crystal structure optimization was carried out using a uniform 4 × 4 × 2 *k*-point grid in the Brillouin zone. The wave function energy cutoffs were configured to 450 eV. The 2D perovskites atomic positions were thoroughly optimized to the point where the Hellman-Feynman forces on each atom fell below 0.01 eV/Å. The transport properties were evaluated using DFT in conjunction with the nonequilibrium Green’s function (NEGF) approach, as executed within the QuantumATK package. The 1 × 4 × 1 supercell was used and the *k*-point mesh is 2 × 1 × 100 for the central region. The real-space mesh cutoff is set to 155 Hartree. The exchange-correlation potential is characterized using the GGA framework with the double zeta polarized (DZP) basis set. The transmission coefficients $T(E)\text{}$ are represented as: $T(E)={\rm{Tr}}[G(E)\varGamma (E)G\dagger (E)\varGamma (E)]$, where *G*(E) represents the retarded Green’s function and *Γ*(E) denotes the self-energy broadening.

## Supplementary information

Supplemental material for 'Real-Space Imaging of Photo-generated Surface Carrier Transport in 2D Perovskites'

## Data Availability

All data supporting the findings of the study can be found within the article, its Supplementary Information, or obtained from the corresponding authors upon reasonable request.
