# Supplementary material for: Real-space imaging of photo-generated surface carrier transport in 2D perovskites
Source: Light Sci Appl. 2025 Mar 18;14:124. doi: 10.1038/s41377-025-01758-5 (PMC11920587; doi:10.1038/s41377-025-01758-5)
Supplement: Supplementary file 1 — Supplemental material for 'Real-Space Imaging of Photo-generated Surface Carrier Transport in 2D Perovskites' [file 41377_2025_1758_MOESM1_ESM.docx]

**Supplementary Information**

Real Space Imaging of Photo-generated Surface Carrier Transport in 2D Perovskites

Lijie Wang^1^, Wentao Wu^1^, Jie Yang^2^, Razan Nughays^1^, Yifan Zhou^3^, Esma Ugur^4^, Xi Zhang^1^, Bingyao Shao^4^, Jian-xin Wang^1^, Jun Yin^3^, Stefaan De Wolf^4^, Osman M. Bakr^5^ & Omar F. Mohammed^1,5*^

^1^Advanced Membranes and Porous Materials Center (AMPM), Division of Physical Science and Engineering, King Abdullah University of Science and Technology, Thuwal 23955-6900, Saudi Arabia.

^2^Key Laboratory of Material Physics, Ministry of Education, School of Physics and Microelectronics, Zhengzhou University, Zhengzhou, 450001, China.

^3^Department of Applied Physics, The Hong Kong Polytechnic University, Kowloon 999077 Hong Kong, China.

^4^KAUST Solar Center (KSC), Division of Physical Science and Engineering (PSE), King Abdullah University of Science (KAUST), Thuwal 23955-6900, Saudi Arabia.

^5^KAUST Catalysis Center (KCC), Division of Physical Sciences and Engineering, King Abdullah University of Science and Technology, Thuwal 23955-6900, Saudi Arabia.

Email: [*omar.abdelsaboor@kaust.edu.sa*](mailto:omar.abdelsaboor@kaust.edu.sa);

**Supplementary Note S1: The dielectric anomalies below the bandgap**

The dielectric anomalies are particularly obvious below the bandgap (BG) for all three samples at around 2.33, 2.15, and 2.05 eV. The absorption data presented in our study were derived by fitting the measured spectroscopic ellipsometric data. The fitting process was performed using an isotropic "B-spline" model that simultaneously fits all three sets of measured data, corresponding to different angles of 65°, 70°, and 75°. Therefore, the quality of the data is heavily reliant on the accuracy of the fitting procedure (as shown in Figure S3). Although the overall fitting appears satisfactory with a mean squared error (MSE) of 2.106, the onset of the "Delta" feature, indicated by the green curve (in Figure S3, the dots are the experimental data, and the solid lines are the fits), poses challenges for accurate fitting. This difficulty arises due to the sharp drop in intensities occurring right below the BG, where the values approach zero. To obtain the best possible fitting results, we employed the "node spacing spectral ranges" method with spectral resolution as high as 0.01 eV within the spectral range of 1.50 to 2.70 eV. Despite these efforts, the fitting-induced anomalies primarily manifest below the BG (Fig. S4). We want to emphasize that these fluctuations are fitting-related artifacts and do not invalidate the key findings and conclusions presented in our study.

**Supplementary Note S2: The band assignments in transient reflection**

In TA measurements, the ΔA signal mainly depends on the ratio of the intensity of transmitted probe light with and without pump excitation, assuming that the loss of transmitted probe light is solely resulting from the sample absorption (assuming that the TA signal is proportional to the change in absorption, ΔT/T∝ΔA). In transient reflection (TR) measurements, which share the same experimental setup, the TR signal (ΔR/R) can be determined by the ratio of the intensity of reflected probe light with and without pump excitation. However, unlike TA measurements that primarily probe the bulk property of the samples, the TR signal mainly detects variations in photo-induced reflection due to the changes in the refractive index at the sample surface.

$$\Delta R=-\lg\frac{I_{R-on}}{I_{R-off}}$$

And,

$$R=\frac{{(n-1)}^{2}+k^{2}}{{(n+1)}^{2}+k^{2}}$$

Where *n* and *k* represent the real and imaginary parts, respectively, of the complex refractive index *ñ*=*n*+i*k*. Carrier-induced optical effects cause small changes in reflection coefficient *R*. Therefore, the change in reflectance, ∆R, can be described in terms of the complex index of refraction. Since ∆R is very small, it can be approximately expressed in a linear form by considering $\Delta n$ and $\Delta k$ as:

$$\Delta R=\frac{\partial R}{\partial n}\Delta n+\frac{\partial R}{\partial k}\Delta k$$

The relation between $\Delta n$ and the $\Delta k$ is essential for interpreting the signal of the differential reflectivity. Assuming that the magnitude of *n* is significantly larger than *k*, the photo-induced ∆R is dominated by $\Delta n$, rather than $\Delta k$. This is in contrast to photo-induced ΔA, which is predominantly influenced by *∆k*.

Therefore, we take the n=1 sample as an example and conducted global lifetime analysis (GLA) to derive decay-associated spectra (DAS) capable of discerning spectral components at specific time delays. As illustrated in Fig. 2i, DAS1 corresponds to a lifetime of ~0.25 ps, representing the spectral characteristics immediately after photoexcitation. At this time delay, the material system remains relatively cool, unaffected by lattice heating-induced changes in the refractive index. Consequently, DAS1 can be attributed to the Δ*k* induced by photoexcitation. Moreover, the presence of positive wings on both the high- and low-energy sides of the negative signal further suggests that DAS1 is associated with changes in absorption rather than refractive index (Fig. 2g and h). Furthermore, the transition from DAS1 to DAS2 reveals the disappearance of the positive wing on the low-energy side, indicating a dominance of Δ*n* in DAS2. Additionally, the red shift in the negative peak position aligns with the temperature-induced differential absorption and refractive index trends, suggesting that DAS1 serves as an indicator of transient absorption changes, while DAS2 is primarily influenced by refractive index alterations.

The assignment of spectral bands can be deduced from DAS1. The negative signal at ~2.45 eV is attributed to the bleaching of the excitonic feature caused by phase-space filling (PSF). Conversely, the positive feature with a peak on the high-energy side can be attributed to excited-state absorption (ESA) of the photo-generated charged species, while the low-energy side possibly be originated from the photo-induced broadening of the exciton band absorption.

**Supplementary Note S3: Decay-associated spectra and temperature-induced differential absorption and refractive index**

A global analysis could simultaneously examines multiple kinetic traces recorded at different probe energies, enables the extraction of different signal components at specific lifetimes. The GLA was performed using a discrete sum-of-exponentials function:^1^

| $S\left( t,\lambda_{exc},\lambda_{pro} \right)=\sum_{j=1}^{n} A_{j}\left( \tau,\lambda_{exc},\lambda_{pro} \right)\exp\left( -\frac{t}{\tau_{j}} \right)\otimes IRF(t)$ |  |
| --- | --- |

Where the $\tau$’s represent the global lifetimes, and the *A*’s are the amplitudes for each kinetic trace. The detected signals are convoluted with the instrument response function (*IRF*), which is modeled by a polynomial function:^2^

| $IRF(\lambda)=c_{0}+\sum_{i=1}^{n} c_{i}{(\frac{\lambda-\lambda_{c}}{100})}^{i}$ |  |
| --- | --- |

The time zero position at the central wavelength, $\lambda_{c}$ , is given by $c_{0}$. GLA results in the so-called DAS, where the pre-exponential amplitudes for each lifetime component are plotted as a function of probe wavelength, $\lambda_{pro}$. Here we take the n=1 sample as an example performing GLA with a time window of 0-100 ps, and the resulting DAS are presented in Fig. 2i.

To calculate the absorption spectra and the reflectivity from temperature-dependent in-situ spectroscopic ellipsometry, we used the relations^3^:

$\alpha=\frac{4\pi k}{\lambda}, R=\frac{{(1-n)}^{2}+k^{2}}{{(1+n)}^{2}+k^{2}}$,

where *n* and *k* are, respectively, the real and imaginary parts of the complex refractive index $ñ=n+ik$:

$n=\sqrt{\frac{\varepsilon_{1}+\sqrt{\varepsilon_{1}^{2}+\varepsilon_{2}^{2}}}{2}}$, $k=\sqrt{\frac{{-\varepsilon}_{1}+\sqrt{\varepsilon_{1}^{2}+\varepsilon_{2}^{2}}}{2}}$.

The spectral changes are better highlighted by subtracting the absorption and refractive index values measured at high temperatures from that at room temperature (25 ^o^C). The resulting differential changes in absorption (ΔA) and refractive index (Δ*n*) are plotted as a function of energy and temperature in Fig. 2g and h.


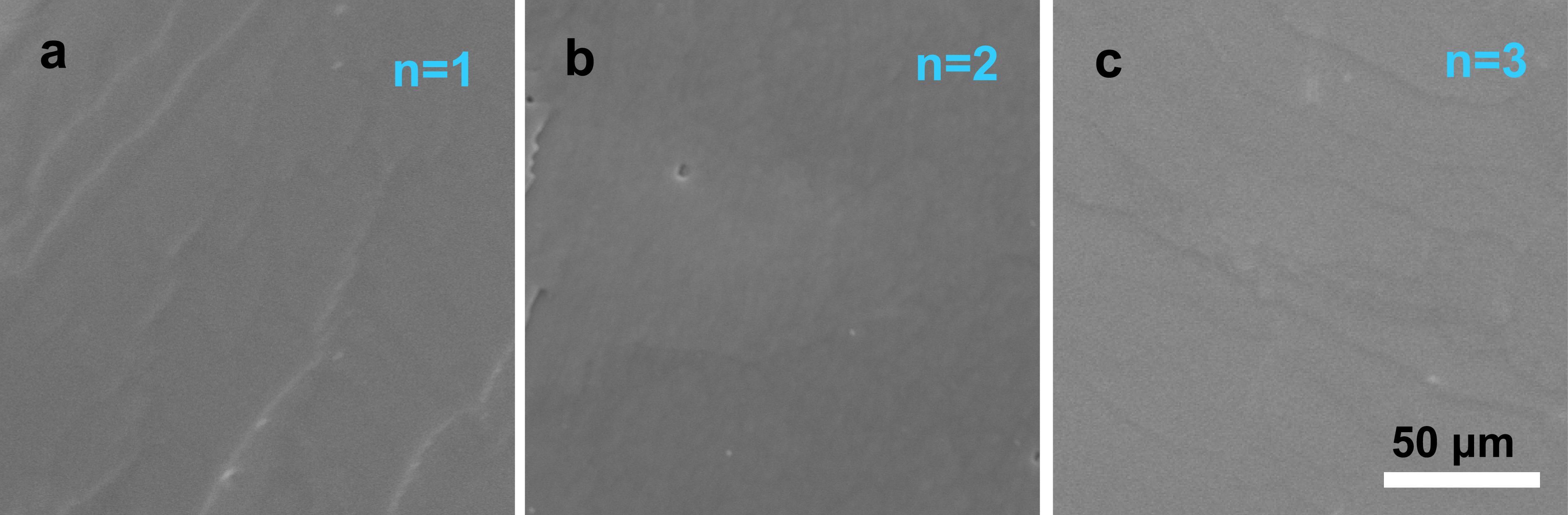


**Fig. S1.** Steady-state SUEM images captured by pulsed electrons in the three measured samples.


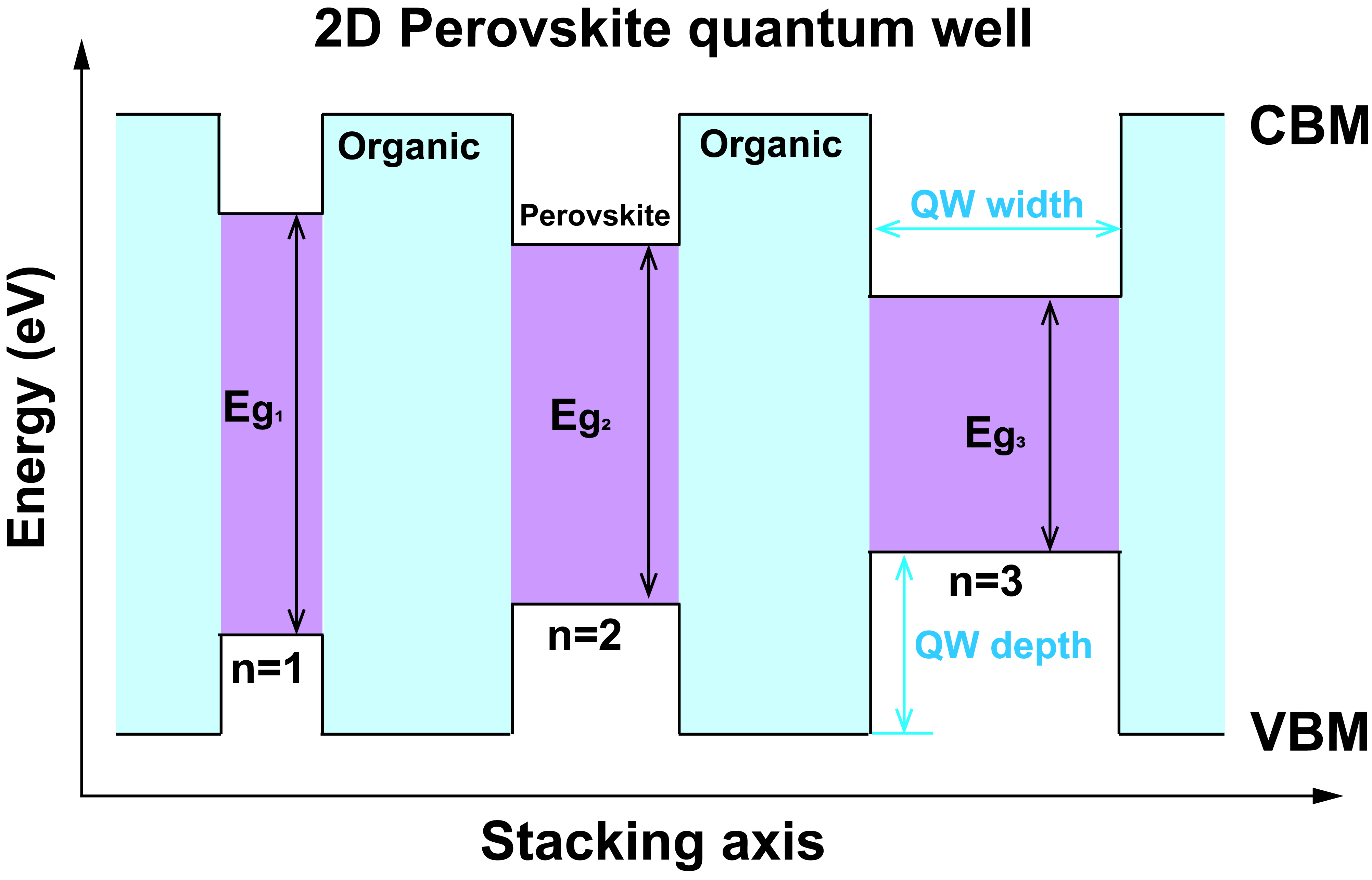


**Fig. S2.** Schematic diagram of QW band structures of 2D perovskites with different n values.


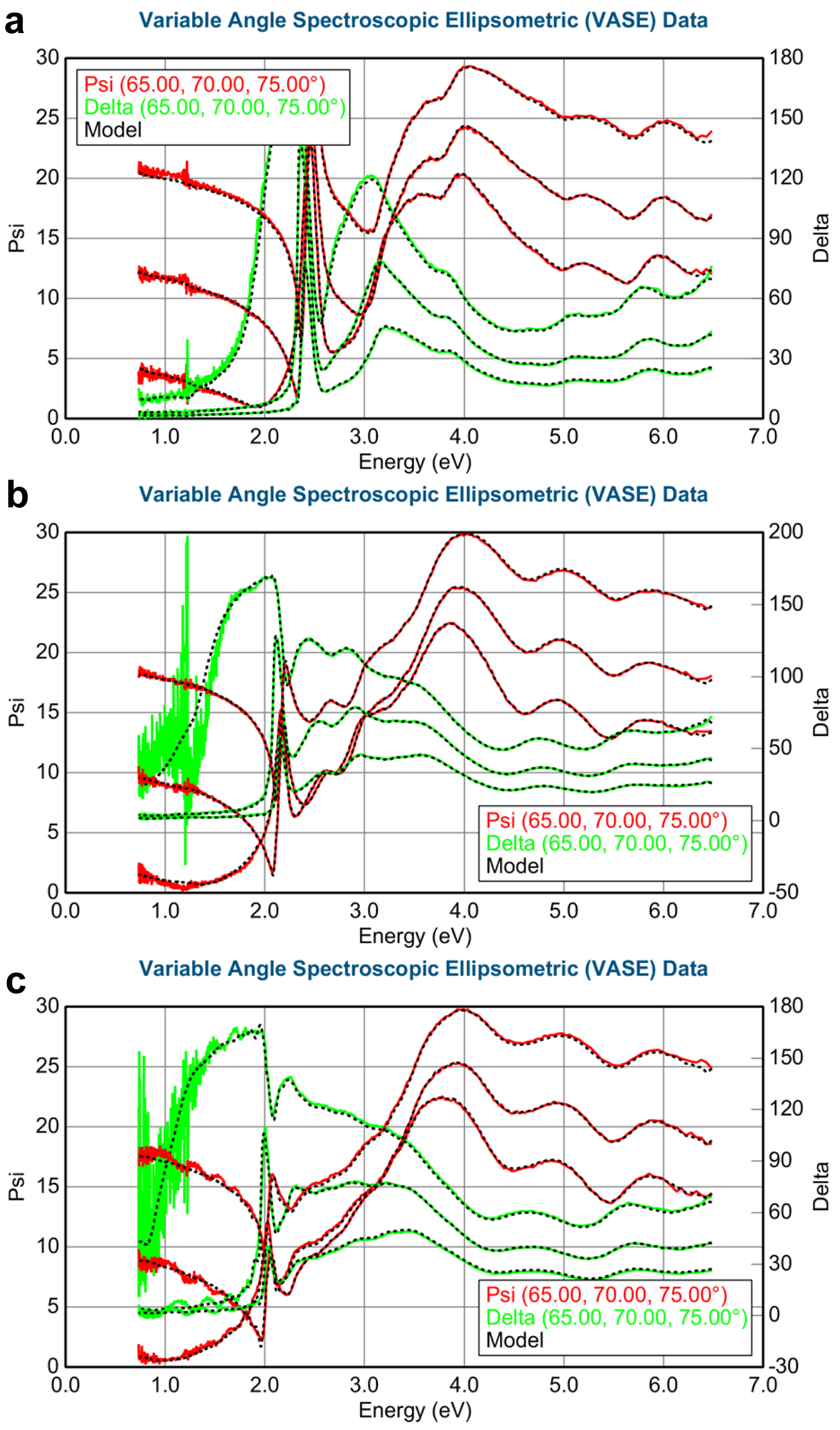


**Fig. S3.** The variable angle spectroscopic ellipsometry data measured at 65°, 70°, and 75°. The dots are the experimental data, and the solid lines are the fits. The dielectric function, absorption and refractive index are extracted based on these fits.


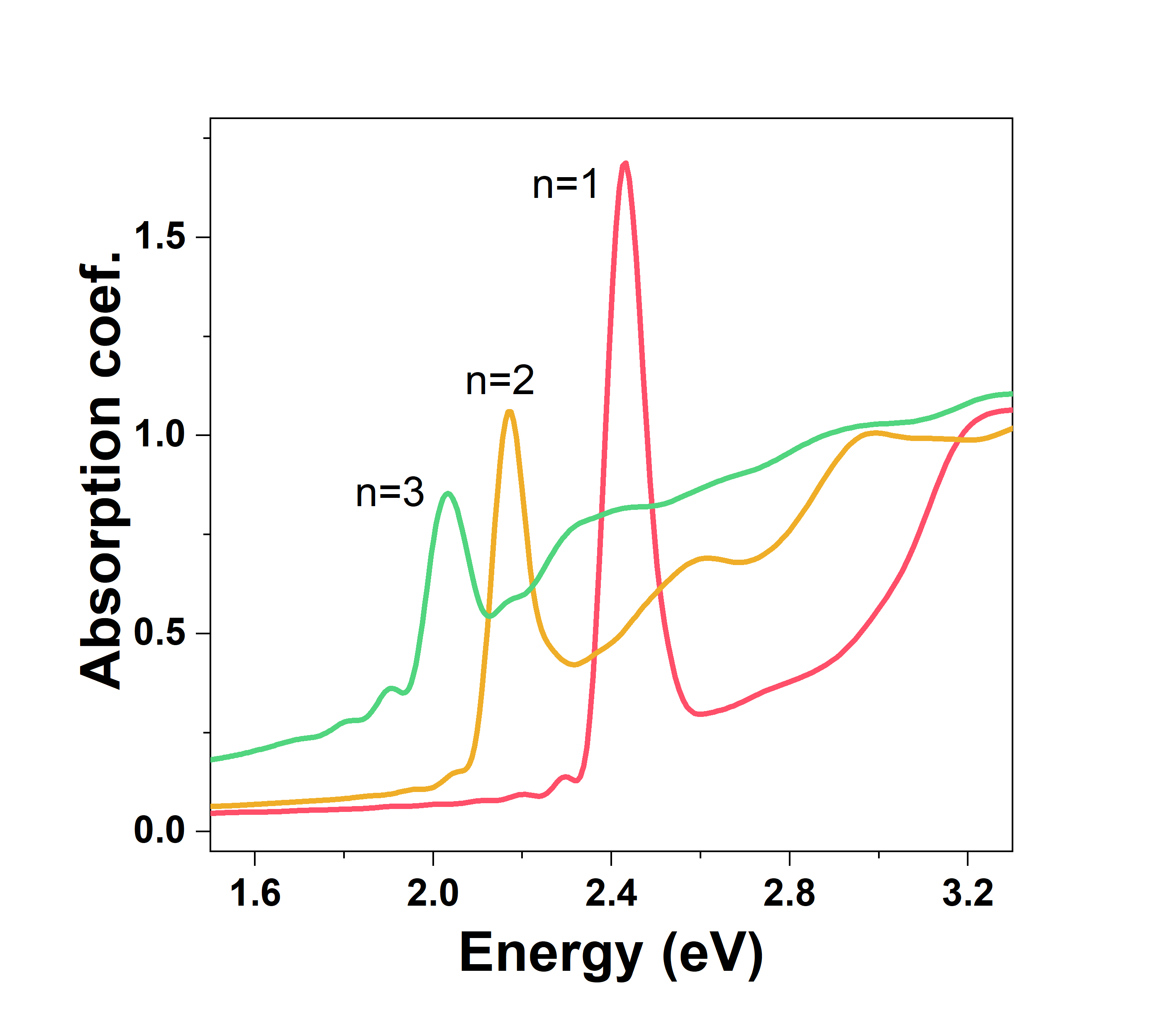


**Fig. S4.** Absorption spectra of the three types of 2D perovskites, obtained by fitting data measured with variable angle spectroscopic ellipsometry.


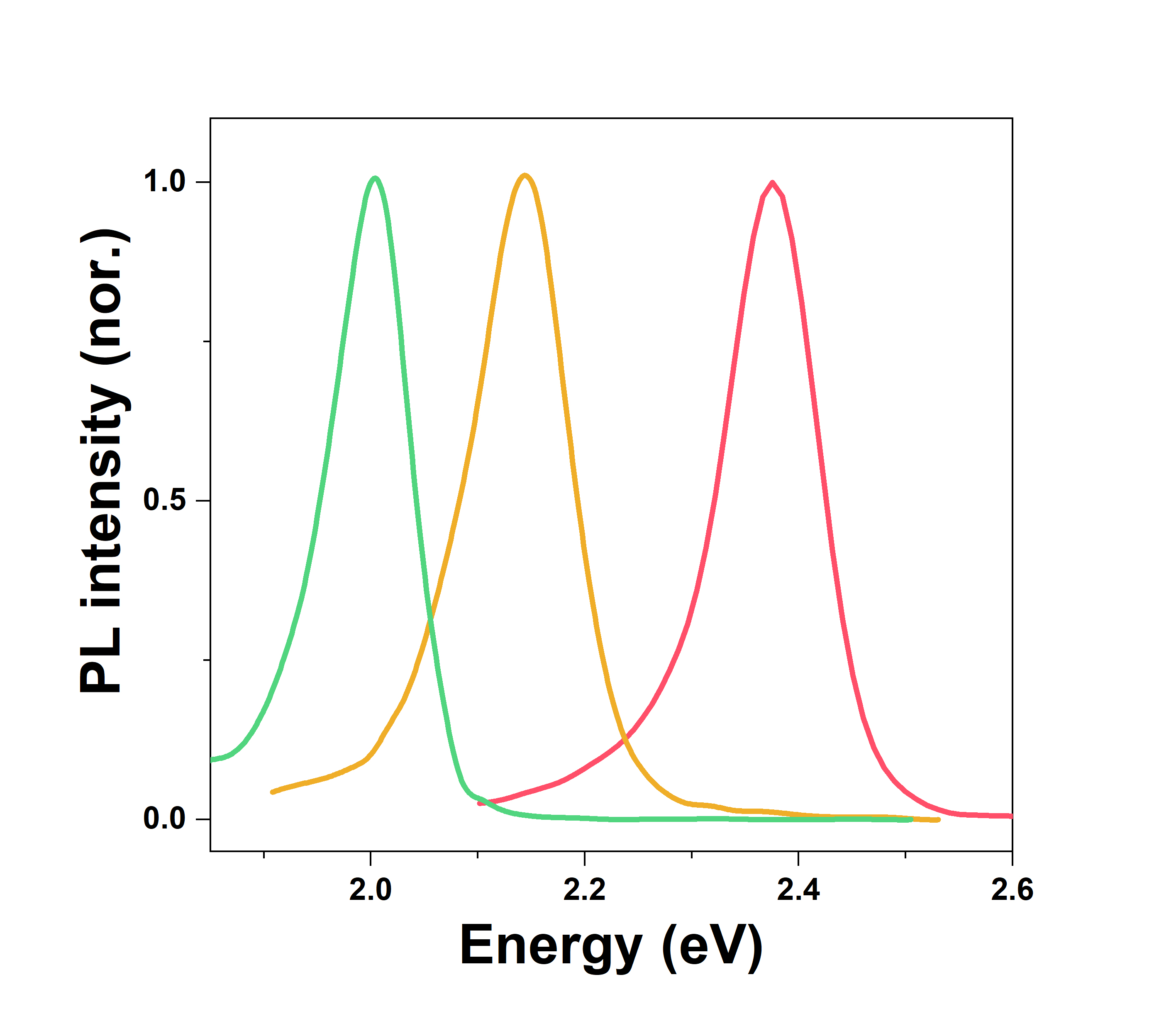


**Fig. S5.** PL spectra of the three prepared crystals; the intensities are normalized for comparison.


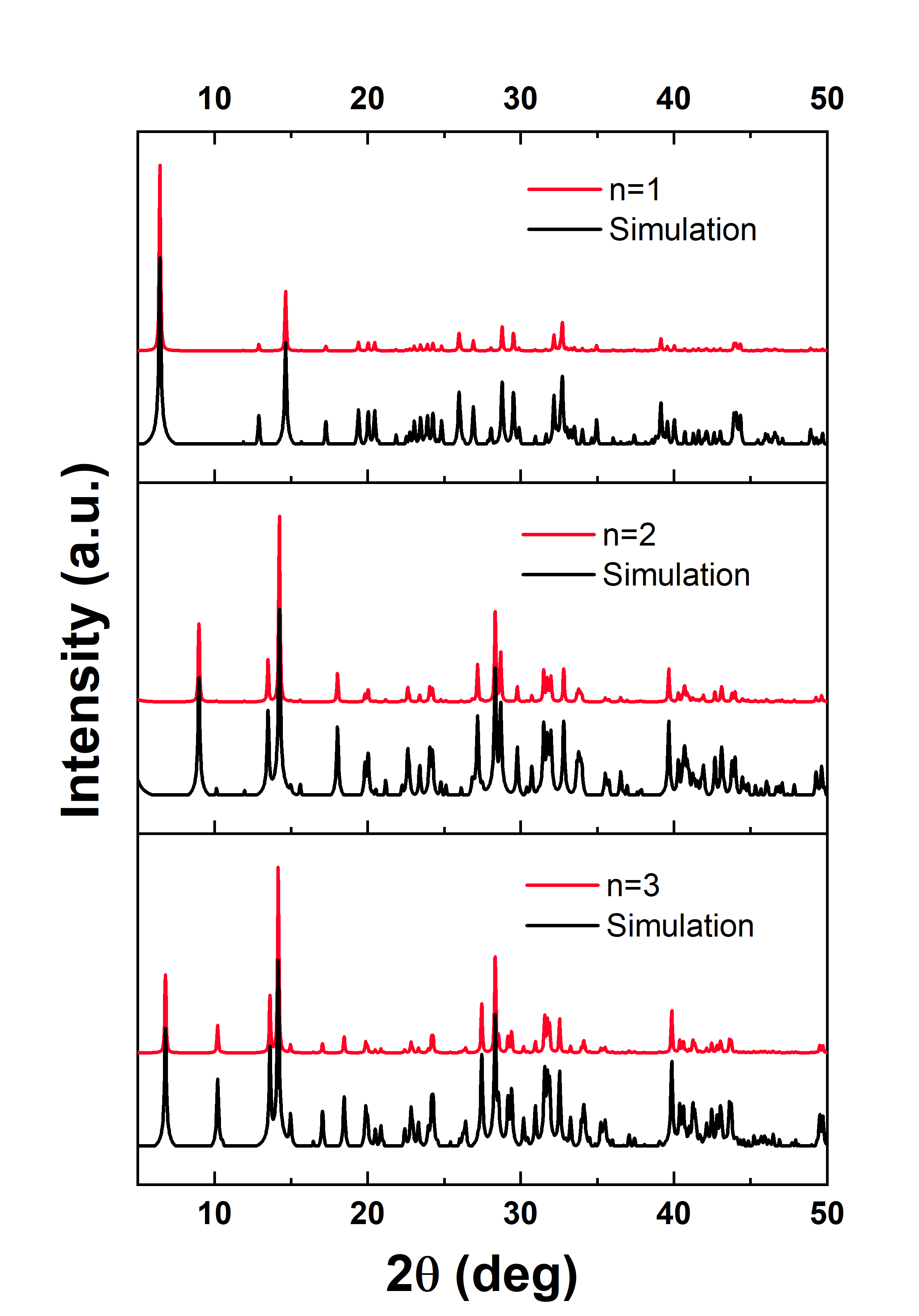


**Fig. S6.** XRD of the three prepared crystals: the red lines represent the measured data, while the black lines indicate the simulations derived from the reported single-crystal X-ray diffraction results from Cambridge Crystallographic Data Centre (CCDC), 665690 (n=1), 1478379 (n=2), 1478380 (n=3).

Given the extensive single-crystal data available in open-source databases, we were able to compare our samples with previously reported structures. A straightforward approach was to compare the experimental XRD patterns of our single crystals with simulated patterns derived from the reported single-crystal X-ray diffraction results from Cambridge Crystallographic Data Centre (CCDC) [e.g., 665690 (n=1), 1478379 (n=2), 1478380 (n=3)]. Matching the experimental XRD peaks with those in the database indicates identical structural characteristics. This is indeed the case, as shown in Fig. S6, where no additional peaks were detected. This comparison sufficiently confirms the accuracy of the stoichiometric formulas and the purity of the samples.


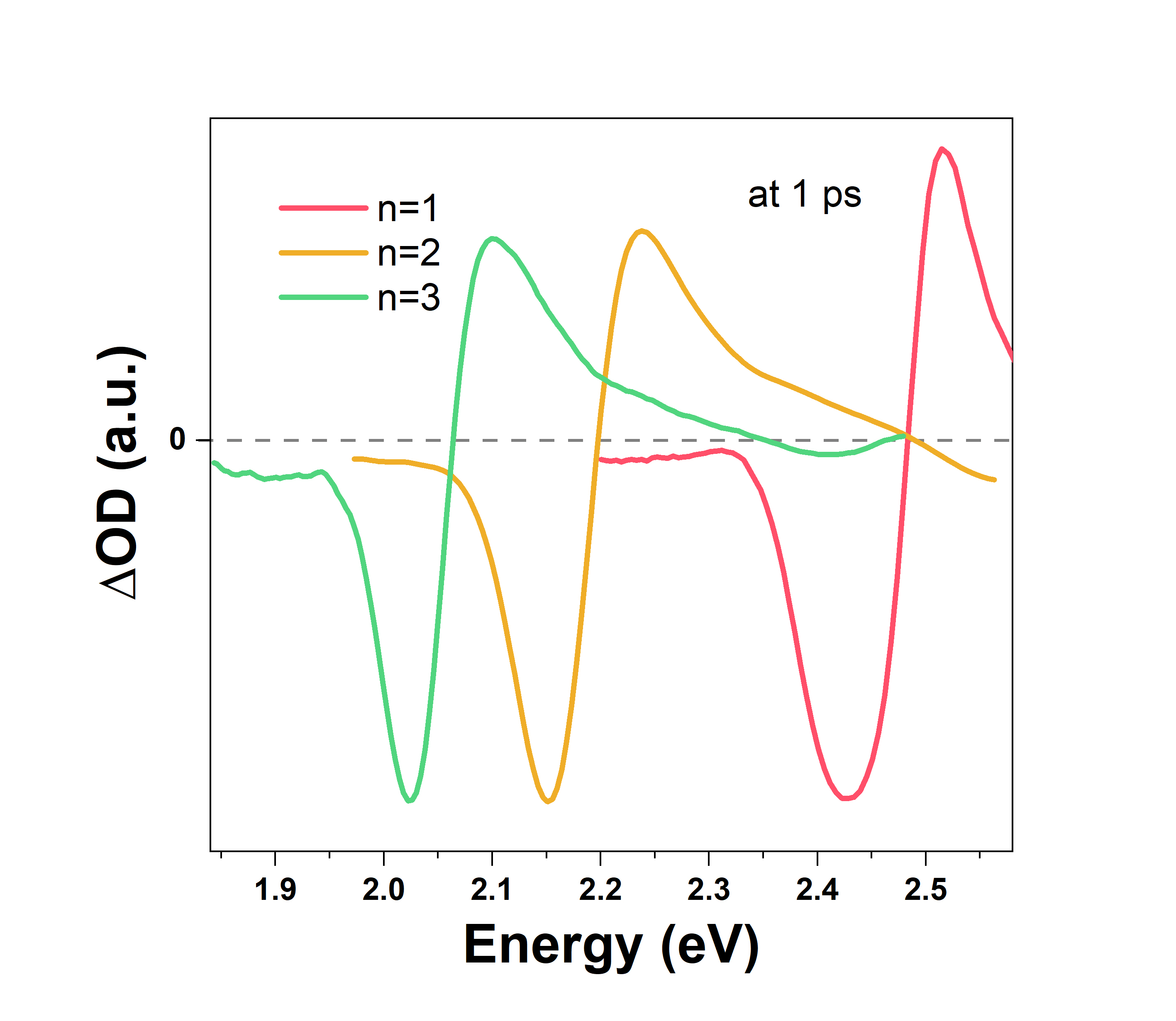


**Fig. S7.** Comparison of the spectral traces recorded at 1 ps for n=1, 2, and 3. All intensities are normalized at the negative maximum position.


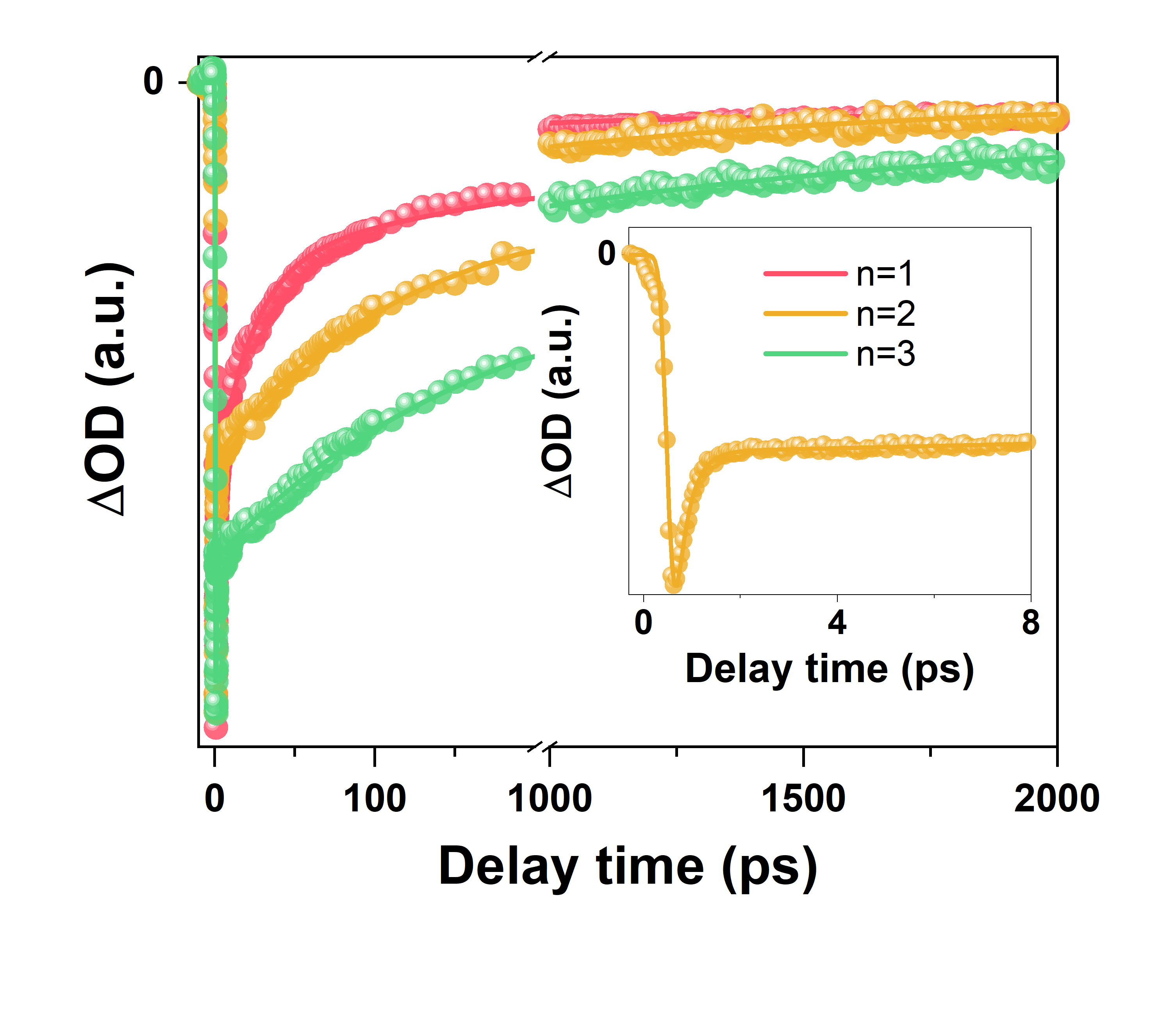


**Fig. S8.** Time traces probed at the maximum of the negative positon of the three measured samples, at the negative signal maximum (at 2.02, 2.18, and 2.43 eV), the dots represent the experimental data, and the solid lines are the fits. All intensities are normalized for comparison. The inset shows the n=2 case zoomed into the first 8 ps.

**Table S1**. Summary of time constants from the three-exponential fitting equation^a^

|  | τ_1_ (ps) | τ_2_ (ps) | τ_3_ (ps) |
| --- | --- | --- | --- |
| n=1 (at 2.43 eV) | 0.2±0.1 | 22.5±4.1 | 341±55 |
| n=2 (at 2.16 eV) | 0.3±0.1 | 104.1±8.2 | 870±89 |
| n=3 (at 2.03 eV) | 0.5±0.1 | 152.2±18.0 | 1111±160 |

a: The fitting functions of three exponential equation:

$$S\left( t \right)=exp{-(\frac{t-t_{0}}{t_{p}})}^{2}*\sum_{i} A_{i}\exp-\left( \frac{t-t_{0}}{t_{i}} \right),$$

$$t_{p}=\frac{IRF}{2\cdot ln2}$$

Where *IRF* is the instrument response function (220±12 fs), *t_0_* is the time zero (140±20 fs), *A_i_* and *t_i_* are amplitudes and decay times respectively, $*$is convolution. In all measurements, the pump fluence is about 50 μJ cm^-2^.


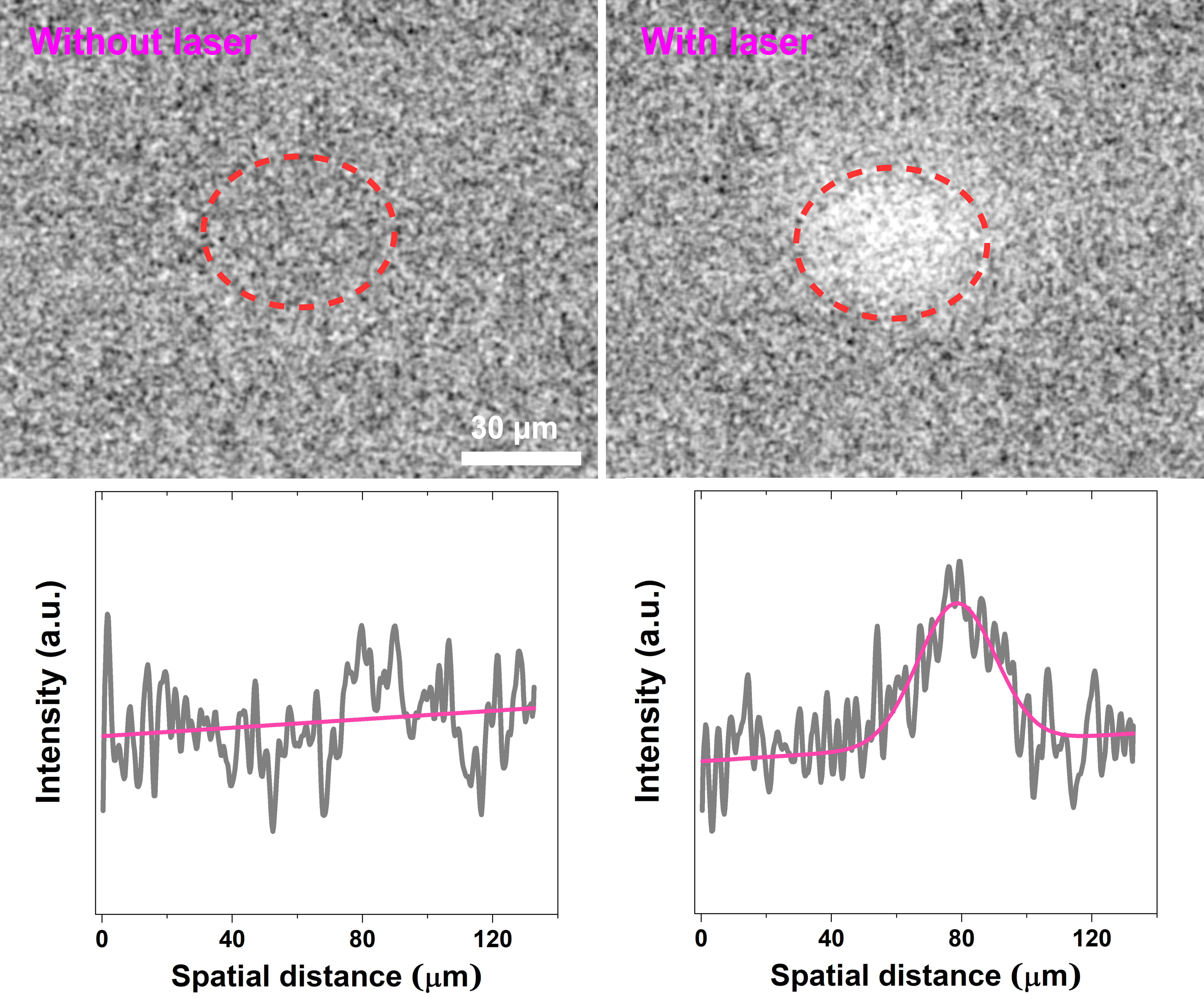


**Fig. S9.** SUEM difference images on a standard silicon wafer sample. The images are filtered using Gaussian blur with a radius of '1.8' pixels, and the contrast is adjusted to enhance the intensity of the bright signal. Upon laser irradiation under identical experimental parameters, the induced spot size is approximately the same size as that of the 2D perovskite at ~2 ps, therefore, we assume the image at ~2 ps as the starting point of the laser footprint in 2D perovskite measurements.

It is worth to note that a higher number of generated electrons at the emitter source can lead to significant broadening of the temporal profile due to electron-electron repulsion, and could lead to a lower resolution than the cross-correlation between the electron probe and the photon pump^4^, in our experiment, with a pulse energy of ~0.4-0.6 nJ that corresponds to ~10-15 electrons per pulse, the temporal resolution of the setup is estimated to be ~1.2±0.3 ps.


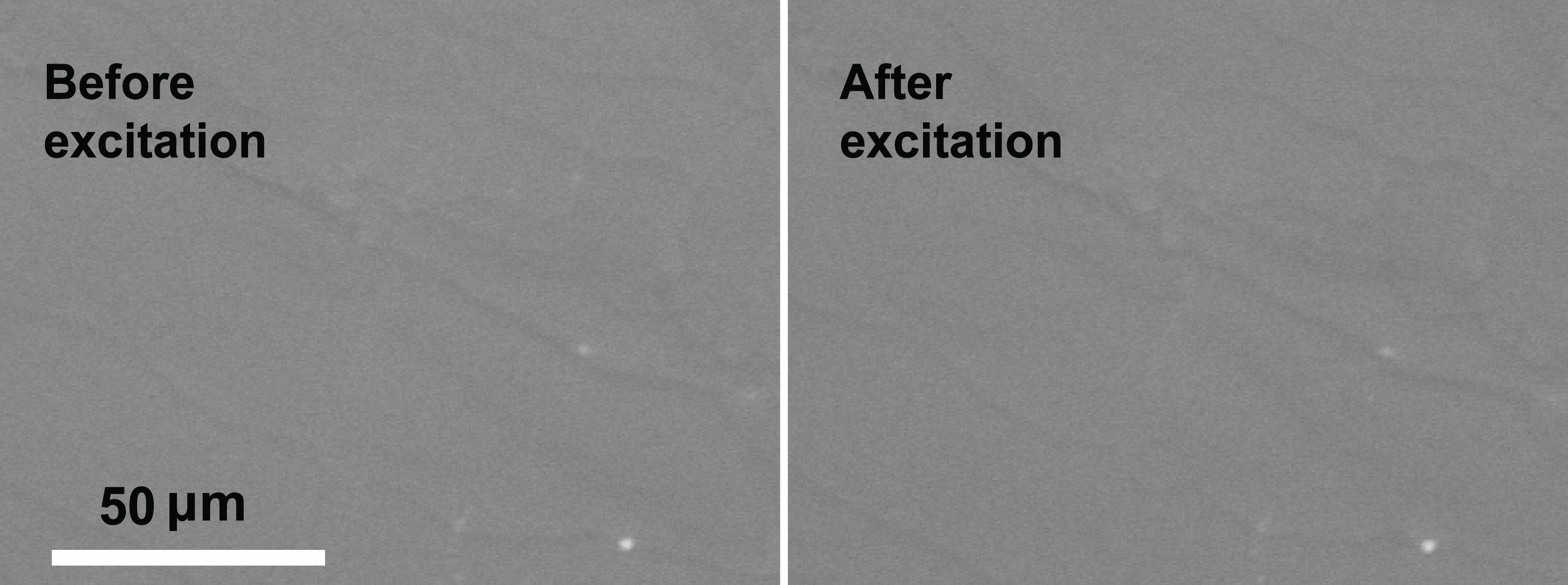


**Fig. S10.** SUEM test before and after photoexcitation on the surface of n=3 2D perovskite.


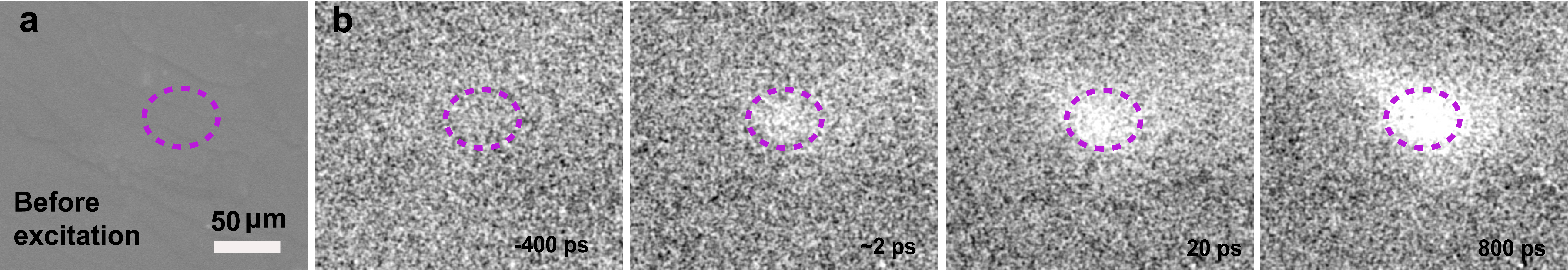


**Fig. S11.** **a**, SUEM image before photoexcitation on the surface of n=3 2D perovskite. The dashed circle represents the laser footprint. **b,** SUEM difference images at -400 ps (far negative), ~2 ps, 20 ps, and 800 ps.


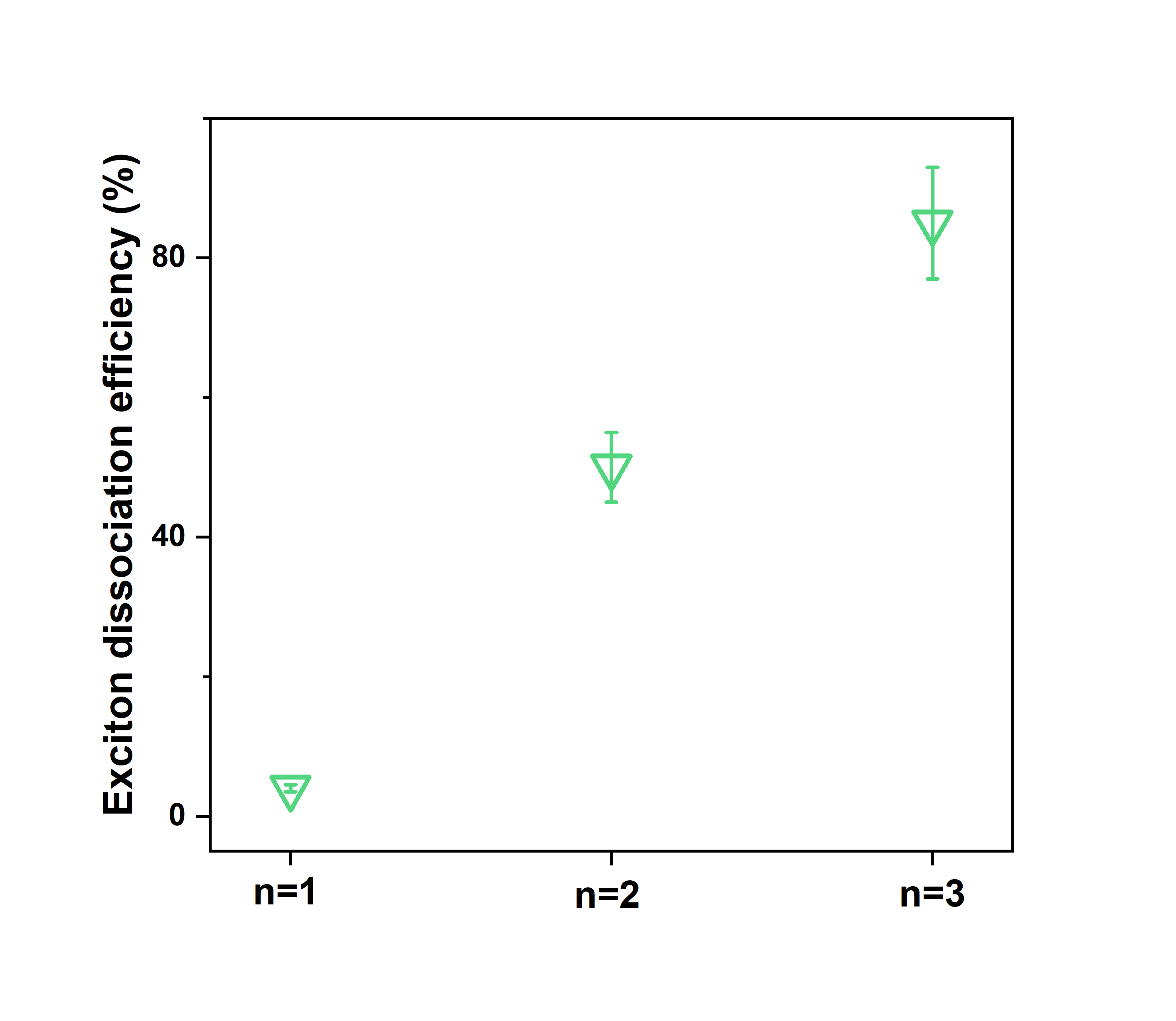


**Fig. S12.** Exciton dissociation rate of the three 2D perovskites.


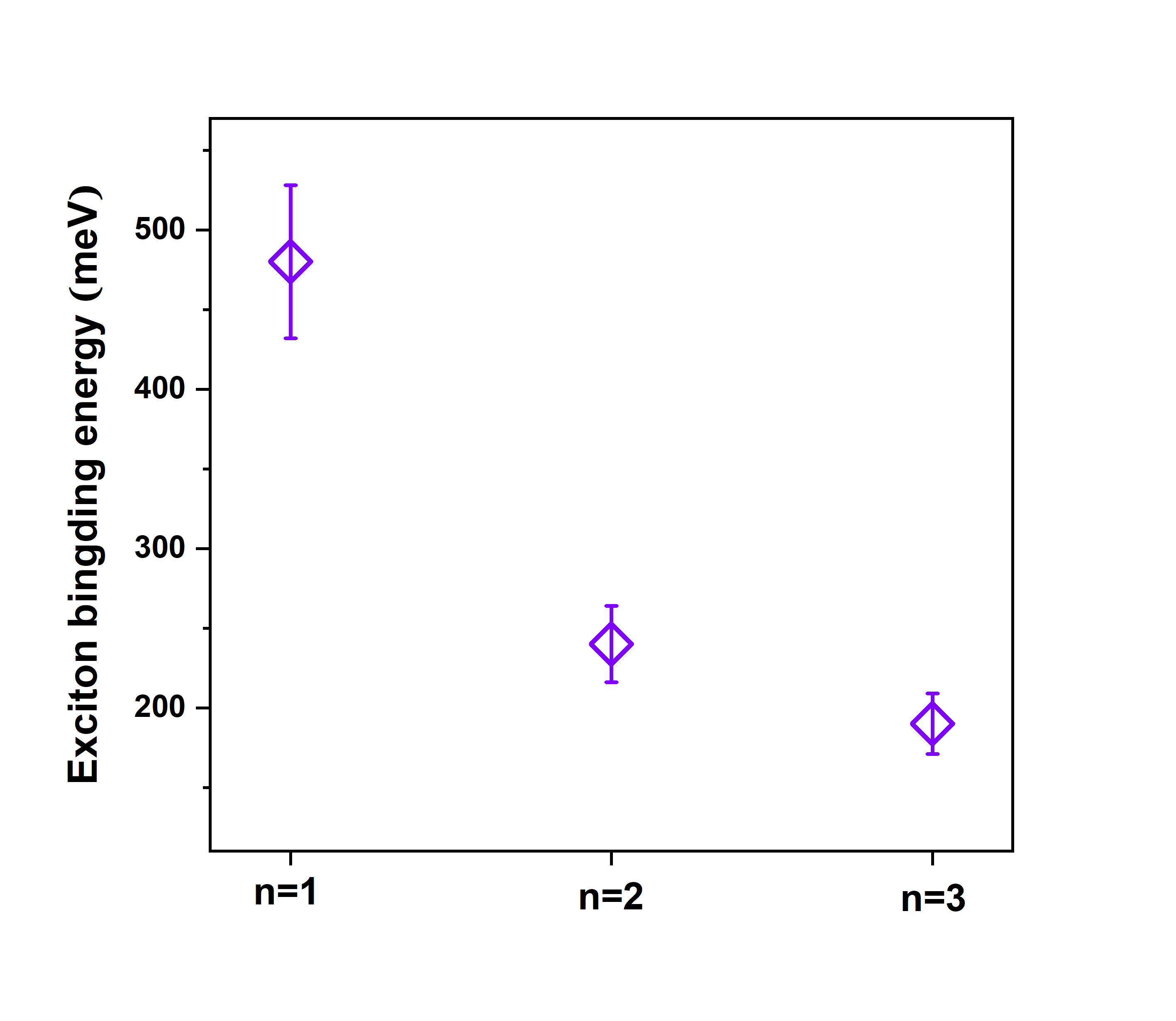


**Fig. S13.** Exciton binding energies of 2D perovskite with respect to the number of layers n.


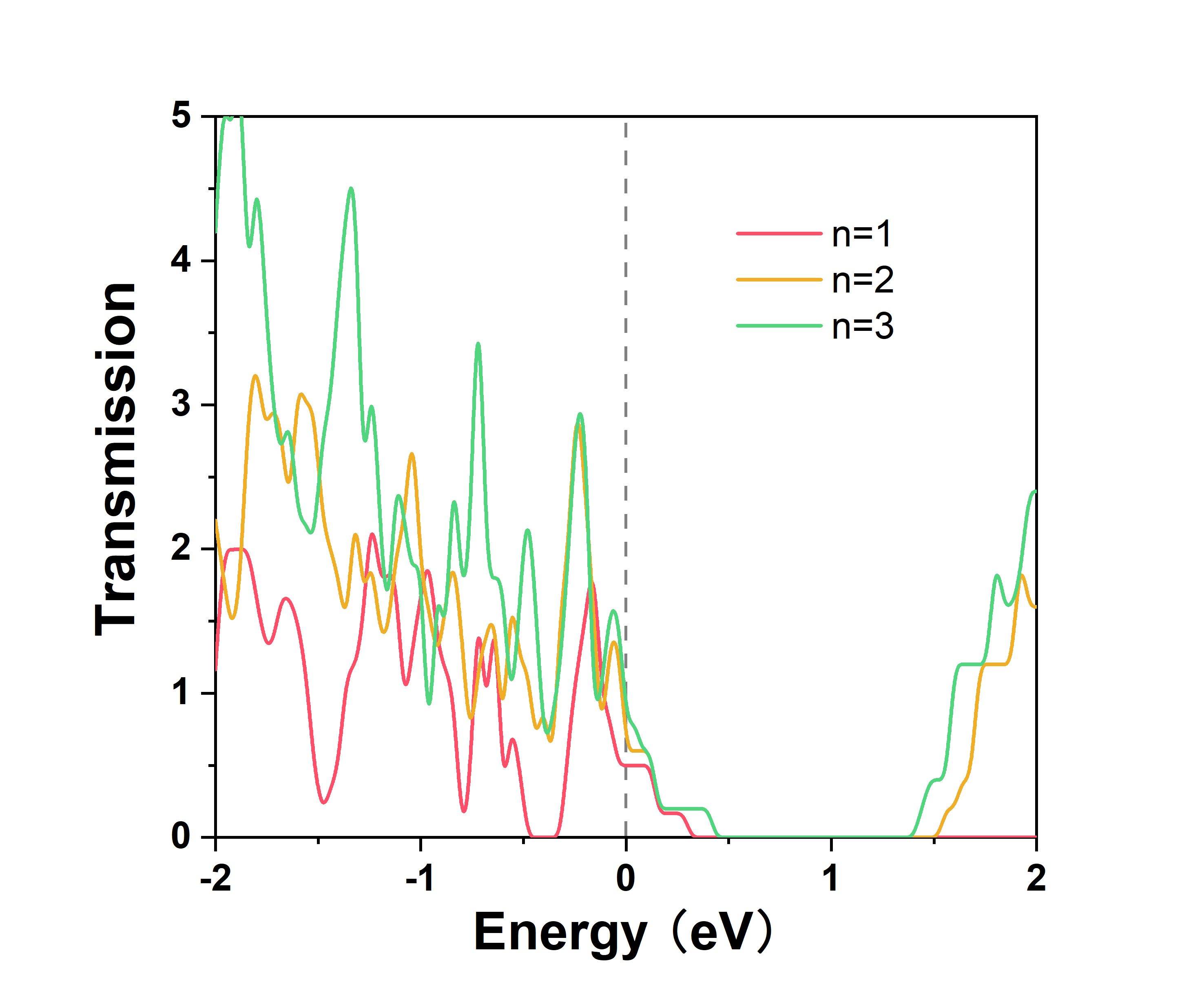


**Fig. S14.** The calculated charge carrier transmission spectra of 2D perovskites. Fermi level is set at zero.

**Table S2.** Comparison of photo-generated carrier/exciton diffusion distances measured by different techniques in layered 2D perovskites, note the different probing timescales in measurements.

| 2D perovskite samples | Methods and probes | Diffusion length (n=1) | Diffusion length (n=2) | Diffusion length (n=3) |
| --- | --- | --- | --- | --- |
| (PEA)_2_(MA)_n–1_Pb_n_I_3n+1_ | tr-PL (bulk, carrier)^5^ | - | 3.7 μm | 3.5 μm |
| (BA)_2_(MA)_n–1_Pb_n_I_3n+1_ | tr-PL (bulk, carrier)^6^ | 7 μm | 11 μm | 14 μm |
| (BA)_2_(MA)_n–1_Pb_n_I_3n+1_ | TAM (bulk, exciton)^7^ | 0.18 μm | 0.25 μm | 0.3 μm |
| (BA)_2_(MA)_n–1_Pb_n_I_3n+1_ | 4D-SUEM (surface, carrier), this work | 3.1 μm | 7.6 μm | 12.3 μm |

**Table S3.** Effective masses of electron and hole along different *k*-path for (BA)_2_PbI_4_, (BA)_2_(MA)Pb_2_I_7_, and (BA)_2_(MA)_2_Pb_3_I_10_ obtained from DFT calculations at GGA/PBE+vdW level.

| **Compounds** | ***k*-Path** | **Electron Effective Mass**  **(×m_0_)** | **Hole Effective Mass**  **(×m_0_)** |
| --- | --- | --- | --- |
| **(BA)_2_PbI_4_** | Γ → X | 0.214 | 0.370 |
|  | Γ → Y | 0.276 | 0.381 |
| **(BA)_2_(MA)Pb_2_I_7_** | Γ → F | 0.244 | 0.353 |
|  | Γ → B | 0.251 | 0.360 |
| **(BA)_2_(MA)_2_Pb_3_I_10_** | Γ → Y | 0.190 | 0.292 |
|  | Γ → Z | 0.193 | 0.282 |

**Table S4.** Summary of 2D perovskite-based solar cell device performance.

| Device | J_sc_ (mA cm^-2^) | Efficiency (%) | Reference |
| --- | --- | --- | --- |
| n=1 (BA)_2_PbI_4_ | 0.06 | 0.01 | ref.^8^ |
| n=2 (BA)_2_(MA)Pb_2_I_7_ | 1.50 | 0.39 | ref.^8^ |
| n=3 (BA)_2_(MA)_2_Pb_3_I_10_ | 9.42 | 4.02 | ref.^8^ |
| n=3 (BA)_2_(MA)_2_Pb_3_I_10_ | 14.37 | 11.44 | ref.^9^ |
| n=3 (BA)_2_(MA)_2_Pb_3_I_10_ | 12.1 | 7.33 | ref.^10^ |
| n=1 (BA)_2_PbI_4_ | 0.07 | 0.01 | ref.^11^ |
| n=2 (BA)_2_(MA)Pb_2_I_7_ | 3.03 | 0.53 | ref.^11^ |
| n=3 (BA)_2_(MA)_2_Pb_3_I_10_ | 6.58 | 1.76 | ref.^11^ |
| n=2 (BA)_2_(MA)Pb_2_I_7_ | 11.0 | 3.5 | ref.^12^ |
| n=3 (BA)_2_(MA)_2_Pb_3_I_10_ | 13.2 | 8.5 | ref.^12^ |

Table S4 provides a summary of efficiencies for same types of 2D perovskite solar cells from various published studies. Although specific values vary across publications, the overall performance trend with different inorganic perovskite layer thicknesses (n = 1, 2, and 3) is consistent with our findings. Specifically, the efficiency of solar cell devices increases significantly from n=1 to n=2, and further to n=3.

**References**

1. Slavov, C. et al. Implementation and evaluation of data analysis strategies for time-resolved optical spectroscopy. *Analytical chemistry* **87**, 2328–2336 (2015).

2. van Stokkum, I.H. et al. Global and target analysis of time-resolved spectra. *Biochimica et Biophysica Acta (BBA) - Bioenergetics* **1657**, 82–104 (2004).

3. Palmieri, T. et al. Mahan excitons in room-temperature methylammonium lead bromide perovskites. *Nature Communications* **11**, 1–8 (2020).

4. Sun, J. et al. Real-space imaging of carrier dynamics of materials surfaces by second-generation four-dimensional scanning ultrafast electron microscopy. *The Journal of Physical Chemistry Letters* **6**, 3884–3890 (2015).

5. Zhao, C. et al. Trap-Enabled Long-Distance Carrier Transport in Perovskite Quantum Wells. *Journal of the American Chemical Society* **142**, 15091–15097 (2020).

6. Shrestha, S. et al. Long carrier diffusion length in two-dimensional lead halide perovskite single crystals. *Chem* **8**, 1107–1120 (2022).

7. Deng, S. et al. Long-range exciton transport and slow annihilation in two-dimensional hybrid perovskites. *Nature Communications* **11**, 664 (2020).

8. Cao, D. H. et al. 2D Homologous Perovskites as Light-Absorbing Materials for Solar Cell Applications. *Journal of the American Chemical Society* **137**, 7843–7850 (2015).

9. Tsai, H. et al. High-efficiency two-dimensional Ruddlesden–Popper perovskite solar cells. *Nature* **536**, 312–316 (2016).

10. Hu, S. et al. Excitonic Solar Cells Using 2D Perovskite of (BA)_2_(FA)_2_Pb_3_I_10_. *The Journal of Physical Chemistry C* **125**, 2212–2219 (2021).

11. Kheirabadi, H. et al. Boosting the Graded Structure of 2D Perovskite Solar Cell Based on BA_2_MA*_n_*_–1_Pb*_n_*I_3_*_n_*_+1_  by Noninteger *n* Values. *ACS Applied Energy Materials* **4**, 394–403 (2021).

12. Ma, C., S. et al. 2D Perovskites with Short Interlayer Distance for High‐Performance Solar Cell Application. *Advanced Materials* **30**, 1800710 (2018).
